# WNT Signaling Factors as Potential Synovial Inflammation Moderators in Patients with Hip Osteoarthritis

**DOI:** 10.3390/biomedicines13040995

**Published:** 2025-04-19

**Authors:** Ivana Jurić, Nela Kelam, Anita Racetin, Natalija Filipović, Davor Čarić, Matko Rošin, Katarina Vukojević

**Affiliations:** 1 Department of Emergency Medicine, University Hospital of Split, Spinciceva 1, 21000 Split, Croatia; ivanajuric55555@gmail.com; 2 Department of Anatomy, Histology and Embryology, University of Split School of Medicine, Soltanska 2, 21000 Split, Croatia; nela.kelam@mefst.hr (N.K.); amuic@mefst.hr (A.R.); natalija.filipovic@mefst.hr (N.F.); 3 Surgery Department, Orthopaedics and Traumatology Division, University Hospital of Split, Spinciceva 1, 21000 Split, Croatia; dcaric@kbsplit.hr (D.Č.); mrosin@kbsplit.hr (M.R.); 4 Center for Translational Research in Biomedicine, University of Split School of Medicine, Soltanska 2, 21000 Split, Croatia; 5 Mediterranean Institute for Life Sciences, University of Split, Meštrovićevo Šetalište 45, 21000 Split, Croatia

**Keywords:** osteoarthritis, synovial membrane, WNT5a/b, β-catenin, acetyl-α-tubulin, Inversin, Dishevelled 1

## Abstract

**Background:** The main feature of osteoarthritis (OA) is the deterioration of articular cartilage, but numerous studies have demonstrated the role of synovial inflammation in the early stages of the disease, leading to further progression of OA. The WNT signaling pathway is involved in numerous activities in joint tissue, but there is a lack of evidence considering the role of WNT in OA synovitis. Our research aims to investigate the expression of WNT Family Member 5A/B (WNT5A/B), β-catenin, acetyl-α-tubulin, Dishevelled-1 (DVL-1), and Inversin (INV) in the synovial membrane of osteoarthritis (OA) hips. **Methods:** The immunohistochemical expressions of the aforementioned proteins in the synovial membrane were analyzed and compared with samples of control group participants with fractured femoral necks. **Results:** The immunoexpression of acetyl-α-tubulin was significantly increased in the intima (*p* < 0.0001) and subintima (*p* < 0.0001) of the group with OA compared with the intima and subintima of the control group. At the same time, acetyl-α-tubulin was also more highly expressed in the intima of the OA group than in the subintima of the OA group (*p* < 0.05); we found the same expression pattern in the control group (*p* < 0.0001). The differential analysis of the GEO dataset did not show significant differences between the osteoarthritis (OA) and control groups in the expression of *TUBA1A*. β-catenin was significantly increased in the subintima (*p* < 0.01) of the group with OA compared to the subintima of the control group. WNT expression has significantly higher positivity in the subintima than in the intima, especially in the control group (*p* < 0.01). *WNT5A* and *WNT5B* were significantly down-regulated in OA compared to the control in the differential analysis of the GEO dataset. The expression of INV and DVL-1 in our study and the differential analysis of the GEO dataset did not differ significantly between the osteoarthritis (OA) and control groups. **Conclusions:** Based on our results, we suggest that acetyl-α-tubulin and β-catenin might be involved in synovial membrane inflammation in OA and serve as potential therapeutic targets.

## 1. Introduction

Osteoarthritis (OA) is a chronic degenerative disease that can affect any joint in the body and cause the loss of joint cartilage [1]. OA is the most common form of arthritis, affecting approximately 15% of the population. The predilection sites are the knee and hip joints [2]. The most frequent risk factors for OA can be separated into person-level factors like age, gender, genetics, obesity, and joint-level factors, which include injury and excessive loading of the joints [3]. OA can be divided into two groups: primary OA, comprising joint degeneration of unknown etiology; and secondary OA, which might be associated with post-traumatic or post-surgical conditions, scoliosis, malposition, congenital or malformation of the limb, and some rare causes, such as rickets, Wilson’s disease, hemochromatosis, chondrocalcinosis, pseudogout, acromegaly, and gout [4,5]. OA develops due to mechanical, genetic, and biochemical factors. It progresses in three stages: first, proteolytic enzymes break down the cartilage matrix; second, the cartilage surface erodes and breakdown products enter the synovial fluid; third, synovial cells perform phagocytosis and release anti-inflammatory cytokines [6]. Matrix fragments from proteolytic enzymes cause synovial inflammation, leading to the release of cytokines (IL-1β, TNF-α) that worsen cartilage damage. These cytokines also block collagen and proteoglycan production and increase MMP levels. Anti-inflammatory cytokines (IL-4, IL-10, IL-13) help reduce this inflammatory response [7,8,9,10,11,12,13,14].

Regulatory pathways that affect chondrocyte activity have protective, degenerative, or dual activities in the joint cartilage tissue. The WNT signaling pathway plays an important role in chondrogenesis and is essential for the physiological maintenance of cartilage in humans and animals [15,16]. Disruption in the WNT signaling pathway leads to an increased production of cytokinins and stimulates synovial inflammation, as well as disrupting the balance between osteoblasts and osteoclasts, which leads to subchondral bone sclerosis and osteophyte formation [17,18,19]. WNT Family Member 5A/B (WNT 5A/B) glycoproteins, which are structurally but not functionally similar, are a part of the canonical and non-canonical WNT signaling pathway family. WNT5A inhibits the canonical WNT signaling pathway by promoting beta-catenin degradation, thereby regulating mammalian limb development and suppressing tumor formation [20,21]. It is associated with synovial membrane inflammation in patients with rheumatoid arthritis [22,23,24]. Also, WNT5A has shown its essential role in destroying cartilage via the growth of blood vessels and cell aggregation. WNT5B signals through a non-canonical signaling pathway independent of β-catenin, but it also acts as an antagonist of canonical WNT signaling [25]. The protein expression and mRNA expression of *WNT5B* were increased in the synovium of OA in a rat model [26]. WNT5B is more expressed in women’s trabecular bone than in men with OA [27].

β-catenin, a central protein in the canonical WNT signaling pathway, plays a dual role in cell adhesion and transcription regulation, potentially influencing cartilage degradation [28]. WNT/β-catenin signaling positively affects the proliferation and activation of synovial fibroblasts [29]. Increased proliferation and hypertrophy of chondrocytes can be caused by increased β-catenin signaling [30,31,32,33,34,35,36]. OA cartilage is known to express higher levels of *CTNNB1* mRNA and β-catenin protein than normal cartilage [37]. The inhibition of β-catenin signaling in cartilage cells of Col2a1-ICAT- transgenic mice leads to increased cell apoptosis, which also leads to the destruction of cartilage in joints [38].

Among the three main WNT signaling pathways, the canonical WNT/β-catenin pathway involves the activation of the Dishevelled (DVL) protein. DVL1 affects the nucleus’s transcription factor and MMP promoter, leading to the degradation of extracellular matrix (ECM) in mouse models of OA [39]. Misregulated *DVL1* alters chondrogenic differentiation and promotes chondrocyte apoptosis, thus leading to cartilage damage in Robinow syndrome [40]. So far, DVL1 expression in the synovial membrane in the context of OA has not been investigated.

The Inversin protein (INV) is involved in regulating cell polarity and the functioning of primary cilia. It acts as a molecular switch between different WNT signaling cascades, but also targets DVL1 for degradation by the ubiquitin–proteasome, which inhibits the canonical WNT pathway. The role of cilia is the regulation of mechanotransduction, which is disrupted in OA and causes irregular cellular responses to mechanical stress. However, the relationship between OA and INV has not been extensively studied [41,42,43,44,45].

Acetyl-α-tubulin is a post-translational modification of α-tubulin, the basic component of microtubules. Microtubules maintain cell structure and functions such as cell division, migration, and response to mechanical stress [46,47]. They play an important role in mechanotransduction and in the function and construction of primary cilia, but in OA, the acetylation of α-tubulin is changed, disrupting both processes [48,49,50].

In this study, we aim to investigate the expression levels of key proteins and signaling molecules—WNT5A/B, β-catenin, acetyl-α-tubulin, DVL-1, and INV—in the synovial tissue of patients suffering from hip joint OA. These molecules are critical components of various cellular signaling pathways known to influence inflammation, cell proliferation, and tissue remodeling within the joint environment, all of which are implicated in early OA progression. By comparing the expression levels of these markers in the synovium of OA patients with the synovium of control patients, we aimed to clarify whether and how these molecular pathways contribute to OA pathology. The correlations established may provide new insights into the molecular mechanisms driving synovitis and offer potential targets for therapeutic intervention in OA.

## 2. Materials and Methods

### 2.1. Study Population

The Ethics Committee of University Hospital Split in Split, Croatia, approved the research (protocol code: 500-03/23-01/230; date of approval: 27 November 2023). The research was conducted following the rules of the Declaration of Helsinki. In the clinical part of the research, which was carried out at the Department of Orthopedics and Traumatology of University Hospital in Split, we performed the selection of subjects and the surgical procedures, in which we sampled hip tissue, while the tissue processing and staining of the samples were carried out at the Department of Anatomy, Histology and Embryology, University of Split School of Medicine. All respondents voluntarily decided to participate in the research and signed their consent to participate.

Hip arthroplasty surgery, in which a total endoprosthesis is implanted in the hip joint, was performed on all participants with OA who met the radiological and clinical criteria, including long-term conservative treatment with limited joint function and long-term pain. The operation was performed through a posterolateral approach to the hip with an incision of the small rotators and the posterior capsule, followed by luxation of the joint. The most damaged cartilage zone from the weight-bearing area of the pathological femur was removed in a triangular manner with an oscillating saw (Trauma Reckon Sytem by Synthes, Switzerland). Evident hip dysplasia, evidence of rheumatological diseases, hip fracture, and infection in the patient’s medical history were exclusion criteria for participants from the group with OA. Subjects assigned to undergo hip arthroplasty due to a recent fracture of the femoral neck with no apparent radiological signs of OA (Kellgren–Lawrence score 0–1) and no previous rheumatic or infectious diseases of the hip in their medical history or signs of OA on radiological images were included in the control group. If subjects had positive results for one antibody—Anti-cyclic citrullinated peptides (anti-CCP) or Rheumatoid factor (RF)—which are examined from the blood and specific for rheumatoid arthritis (RA), they were excluded from the study.

The osteoarthritis group participants were diagnosed according to the following measures: Harris Hip Score (HHS), Western Ontario and McMaster Universities Arthritis Index (WOMAC), visual analog scale (VAS), and radiological Kellgren–Lawrence (K-L) rating scale. Both the OA and control group was assessed according to the histological Krenn synovitis score. The study included 34 participants, of whom 10 were assigned to the control group and 24 to the osteoarthritis (OA) group.

### 2.2. Tissue Collection and Basic Staining Procedures

All groups of participants underwent surgery under spinal anesthesia using the posterolateral approach to the hip, in which an incision is made in the posterior capsule to implant a prosthesis in the hip joint to treat fractures or OA. After extraction, synovial tissue samples from the lower part of the femoral neck next to the head of the bone were placed in containers with formalin solution and forwarded to the Department of Anatomy, Histology and Embryology for further processing and analysis.

After fixation, the tissues were embedded in paraffin and cut into 5 µm thick sections [51,52].

### 2.3. Immunofluorescence Staining

First, the histological slides were processed through deparaffinization in xylene. They were exposed to rehydration in graded water and ethanol solutions, heated in 0.01 M citrate buffer (pH 6.0) for 30 min at 95 °C, and cooled at room temperature. After washing the slides with 0.1 M phosphate-buffered saline (PBS), slides were coated with protein-blocking solution (ab64226, Abcam, Cambridge, UK) for 20 min to inhibit nonspecific staining. The slides were incubated overnight with primary antibodies in a humid chamber at room temperature (Table 1). Then, the slides were washed again with PBS and incubated with secondary antibodies for 1 h (Table 1). The slides were washed again with PBS, and the nuclei were stained with DAPI (4′,6-diamidino-2-phenylindole). Mounting media (Immuno-Mount, Thermo Shandon, Pittsburgh, PA, USA) and a coverslip were used to cover the slides [51,52,53].

### 2.4. Data Acquisition and Quantitative Analysis

The slides were examined with a fluorescence microscope (Olympus BX61, Tokyo, Japan) equipped with a Nikon DS-Ri2 camera (Nikon Corporation, Tokyo, Japan) with NIS-Elements F software, (version 5.22.00) which was utilized to capture microphotographs. Ten non-overlapping fields per sample were taken using a 40× objective magnification to analyze the expressions of the green signal, which represents the positively stained proteins Inversin and WNT5A/B, and the red signal, which represents the positively stained proteins acetyl-α-tubulin, DVL-1, and β-catenin. In order to determine the quantitative analysis of the immunoreactivity of the observed proteins, we calculated the area percentage affected by the signal in the microphotographs captured. In the first step, using the Lasso tool in Adobe Photoshop (Adobe, San Jose, CA, USA), the intima of the synovial membrane was separated from the subintima. ImageJ Software, version 1.54 (NIH, Bethesda, MD, USA), was used to isolate the positive signal during image processing, as described previously [51,54,55]. The first step in Image J included duplicating the microphotograph, filtering the red, green, and blue channels, and subtracting the red channel from the original picture. The processed microphotograph was duplicated and a median filter with a radius of 6.0 pixels was applied and subtracted using the image calculator tool. The processed images were thresholded using the triangle method, and using the “analyze particles” function, the percentage of the image surface was determined. To calculate the actual area percentage, we had to correct the area percentage because a large part of the analyzed images lacked tissue. Using the magic wand tool in Photoshop, the total number of pixels and the number of pixels of empty space were determined.

The corrected area percentage was calculated by dividing the uncorrected area percentage multiplied by the total number of pixels less the number of pixels representing empty space, as previously described [51].

### 2.5. Differential Gene Expression Analysis

The National Center for Biotechnology Information’s Gene Expression Omnibus (GEO) database houses datasets from various experiments, allowing users to download gene expression profiles [56]. In our search for GEO datasets with related gene expression profiles, we utilized the keywords “osteoarthritis”, “Homo sapiens”, and “Expression profiling by array”, which resulted in 12 available studies. We selected the GSE55235 series (Rheumatoid arthritis and osteoarthritis: synovial tissues (Berlin dataset)), which includes the gene expression data from 30 samples, featuring 10 samples of synovial tissue from osteoarthritic joint, 10 samples of synovial tissue from rheumatoid arthritis joint, and 10 control samples of synovial tissue from healthy joints matched for age and gender [57]. We analyzed two groups: we discarded the samples from synovial tissue from rheumatoid arthritis since this was not the focus of our research. The data were obtained using 3–5 µg of total RNA, amplified and labelled using GeneChip^®^ one-cycle target labelling and control reagents (Affymetrix). GeneChips were scanned using the Affymetrix GeneChip Scanner 3000 (Affymetrix^®^ Inc., Santa Clara, CA, USA). To analyze the raw gene expression data, we utilized the online statistical tool GEO2R [56]. The Benjamini and Hochberg (false discovery rate) method was used to calculate adjusted *p*-values. The limma precision weights (vooma) function and quantile normalization (limma package version 3.28.14) were applied to the expression data. To identify significantly differentially expressed genes in the dataset, we determined the following criteria: |log2 (fold change)| > 1 and *p* < 0.05. Up-regulated genes were identified with log2FC ≥ 1, while down-regulated genes were determined by log2FC ≤ −1. GraphPad Prism software (version 9.0.0.) and Adobe Photoshop (version 21.0.2) were used to create the volcano plot.

### 2.6. Statistical Analysis

GraphPad Prism version 9.0.0. software (GraphPad Software, San Diego, CA, USA) is a program that statistically analyzes data. The results are presented as the calculated percentages’ mean value ± standard deviation.

The normality of the data related to the socio-demographic characteristics of the patients was assessed using the Shapiro–Wilk test. Since the data did not follow a normal distribution, the Kruskal–Wallis test was used for the analysis presented in Table 2. Every value with a *p* smaller than 0.05 was considered statistically significant (*p* < 0.05).

Two-way analysis of variance (ANOVA) with Tukey’s post hoc test was used to analyze the differences in protein expression between the sample categories. Every value with a *p* smaller than 0.05 was considered statistically significant (*p* < 0.05).

## 3. Results

### 3.1. Sociodemographic Characteristics of Participants

The study included 34 participants, of whom 10 were assigned to the control group and 24 to the osteoarthritis (OA) group. Known data for all participants were body mass index (BMI), gender, and age (Table 2) [51,52].

The mean synovitis score of all participants with OA was 2.59 (range 0–6). Compared to the original Krenn scoring profile for OA, the score for our OA group was higher by 0.59 [58]. Ten participants were included in the control group; the average synovitis score was 0.62 (range 0–2), presenting a statistically significant difference in comparison with the OA group. In the Supplementary Materials, we show a graph in which the OA group is divided into two subgroups according to the Krenn synovitis score: [58] low synovitis score (LSS OA) and higher synovitis score (HSS OA).

### 3.2. Hematoxylin–Eosin Staining of the Synovial Membrane in Patients with Hip Osteoarthritis

**Figure 1:**
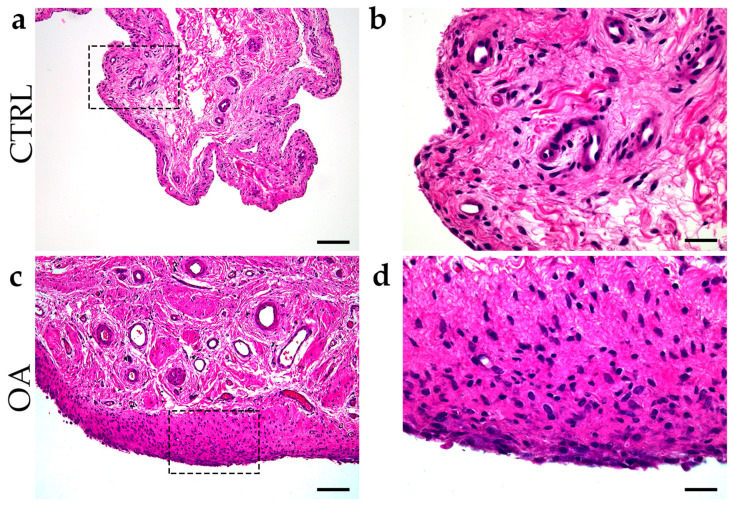
Differentiation between control group and OA group. Images of tissue biopsies show control tissue (CTRL) (**a**,**b**) and synovitis in OA group (**c**,**d**). Images (**c**,**d**) show hyperplasia of the sheath layer, activation of resident cells (stroma), and inflammatory infiltrate (H&E staining). Images (**b**,**d**) indicate enlarged areas marked with dashed boxes (**a**,**c**). Images were taken at magnifications of 4× (**a**,**c**) and 10× (**b**,**d**). The scale bars are 200 µm (**a**,**c**) and 100 µm (**b**,**d**).

Inflammation of the synovial membrane with infiltration of synovial mononuclear cells, hyperplasia of the membrane cells, and an increase in the thickness of the cell layer of the synovial membrane were common characteristics of synovitis in the OA group (Figure 1).

### 3.3. Double Immunofluorescence Staining of Inversin and DVL-1

**Figure 2:**
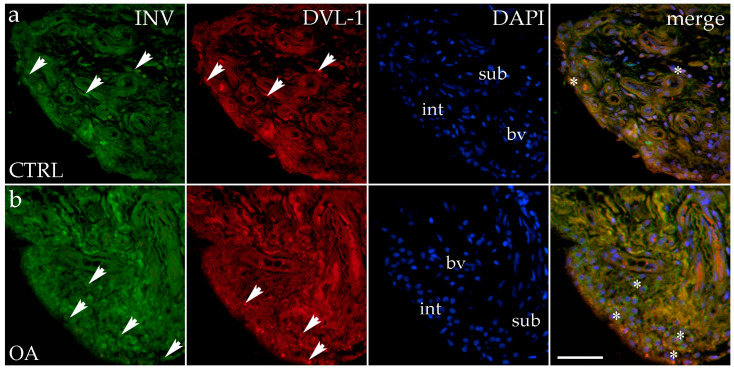
Immunohistochemical staining with Inversin (INV) and Dishevelled-1 (DVL-1) of the synovial membrane in participants with hip osteoarthritis (OA). (**a**) Hip synovium of participants without OA (controls); (**b**) hip synovium of participants with osteoarthritis (OA); int—intima; sub—subintima; bv—blood vessel. INV-positive cells (green signal) can be seen in the intima (arrows) and subintima (arrows) of all analyzed groups (**a**,**b**). DVL1-positive cells (red signal) can be seen in the intima (arrows) and subintima (arrows) of all analyzed groups (**a**,**b**). Strong positivity for INV and DVL-1 can be seen in cells of the subintimal blood vessels of the control group. All cell nuclei represented in blue are stained with 4′,6-diamidino-2-phenylindole (DAPI). The far-right column (merge) represents INV and DVL-1 merged with DAPI nuclear staining. Asterisk denotes the zone where the co-expression was detected. Photos were taken at a magnification of ×40; the scale bar referring to all images is 50 μm. The study included 34 participants, of whom 10 were assigned to the control group and 24 to the osteoarthritis (OA) group.

Immunohistochemical staining with an Inversin marker revealed positive expression in the synovial membranes of patients with hip OA and in the healthy controls. The positive cells were found in the subintimal blood vessels of the control group and the intima and subintima of the OA group. Both markers are co-expressed in intimal and subintimal cells (Figure 2).

**Figure 3:**
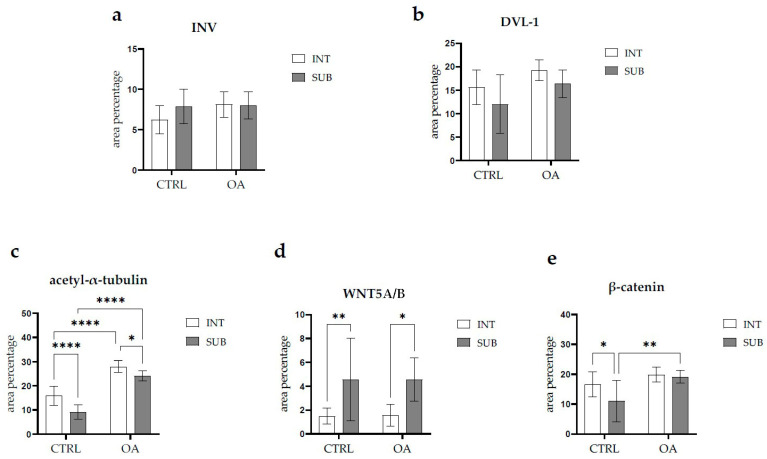
Statistical analyses of protein immunoexpression of (**a**) Inversin (INV), (**b**) Dishevelled-1 (DVL-1), (**c**) acetyl-α-tubulin, (**d**) WNT Family Member 5A/B (WNT5A/B), and (**e**) β-catenin in the synovial membrane of participants with hip osteoarthritis (OA). INT—intima; SUB—subintima; CTRL—controls; OA—group with osteoarthritis. We analyzed the data using two-way ANOVA with Tukey’s post hoc test. The bars of the graphs represent the mean area percentage of the immunofluorescence signal of the analyzed proteins, while the error bars represent the standard deviation. Asterisks mark significant differences: * *p* < 0.05, ** *p* < 0.01, **** *p* < 0.0001. The study included 34 participants, of whom 10 were assigned to the control group and 24 to the osteoarthritis (OA) group.

There were no statistically significant differences in Inversin immunoexpression between the intima and subintima across all analyzed groups (*p* > 0.05) (Figure 3).

There were no statistically significant differences in Inversin immunoexpression between the intima and subintima across all analyzed groups (*p* > 0.05). The only distinction observed among the groups was that Inversin expression was higher in the intima compared to the subintima in the low-synovitis-score group, but without statistical significance (Figure S1a).

DVL-1 displayed positivity in the intima and subintima of patients with hip OA and in the healthy controls. We observed positive cells in the subintimal blood vessels of the control and OA group and the intima and subintima of all groups examined (Figure 2). The intima represented a higher immunoexpression of DVL-1 than the subintima of all analyzed groups, but without statistical significance (Figure 3).

### 3.4. Double Immunofluorescence Staining of WNT5A/B and β-Catenin

**Figure 4:**
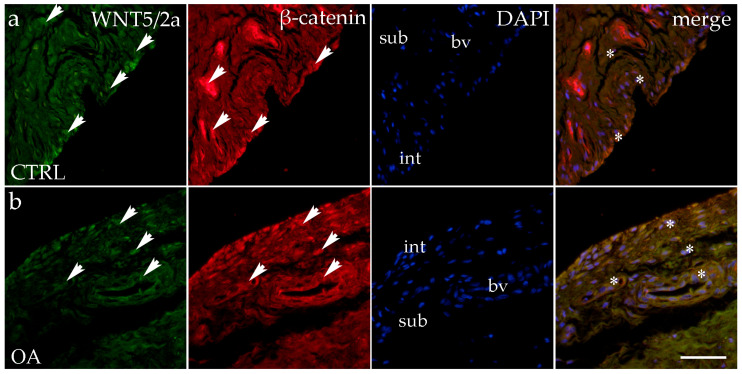
Immunohistochemical staining with WNT Family Member 5a/b (WNT5A/B) and β-catenin in the synovial membrane of participants with hip osteoarthritis (OA). (**a**) Hip synovium of participants without OA (controls); (**b**) hip synovium of participants with OA; int—intima; sub—subintima; bv—blood vessel. WNT5A/B—positive cells (green signal) can be seen in the intima (arrows) and subintima (arrows) of all analyzed groups (**a**,**b**). β-catenin-positive cells (red signal) can be seen in the intima (arrows) and subintima (arrows) of all analyzed groups (**a**,**b**). WNT5A/B and β-catenin strong positivity can be seen in cells of the subintimal blood vessels of the control group. All cell nuclei represented in blue are stained with 4′,6-diamidino-2-phenylindole (DAPI). The far-right column (merge) represents WNT5A/B and β-catenin merged with DAPI nuclear staining. Asterisk denotes the zone where the co-expression was detected. Photos were taken at a magnification of ×40; the scale bar referring to all images is 50 μm. The study included 34 participants, of whom 10 were assigned to the control group and 24 to the osteoarthritis (OA) group.

When analyzing the expression of WNT5A/B in the synovial membrane of participants with hip OA and in the healthy controls, we noticed positive cells in the intima and subintima of all analyzed groups (Figure 4). Strong WNT5A/B positivity can be seen in the subintimal blood vessels of all the analyzed groups. In each analyzed group, we noticed that WNT expression had significantly higher positivity in the subintima than in the intima, especially in the control group (*p* < 0.01) (Figure 3).

The immunoexpression of β-catenin was observed in the synovial membrane of patients with hip osteoarthritis (OA) and in the healthy controls (Figure 4). β-catenin-positive cells were present in both the intima and subintima across all analyzed groups. The intima showed higher positivity than the subintima in all groups. Notably, strong β-catenin positivity was also observed in the subintimal blood vessels of the analyzed groups (Figure 3).

Statistical analysis revealed that in the control group, β-catenin expression was significantly higher in the intima compared to the subintima (*p* < 0.05). Additionally, β-catenin expression in the subintima was considerably higher in the OA group compared to the control group (*p* < 0.01) (Figure 3).

Statistical analysis revealed that in the control group, β-catenin expression was significantly higher in the intima compared to the subintima (*p* < 0.05). Additionally, β-catenin expression in the intima was considerably higher in the low-synovitis-score (LSS) group compared to the control group (*p* < 0.05). Furthermore, β-catenin expression was significantly higher in the subintima of both the LSS group (*p* < 0.001) and the high-synovitis-score (HSS) group (*p* < 0.05) compared to the subintima of the control group (Figure S1b).

### 3.5. Immunofluorescence Staining of Acetyl-α-Tubulin

**Figure 5:**
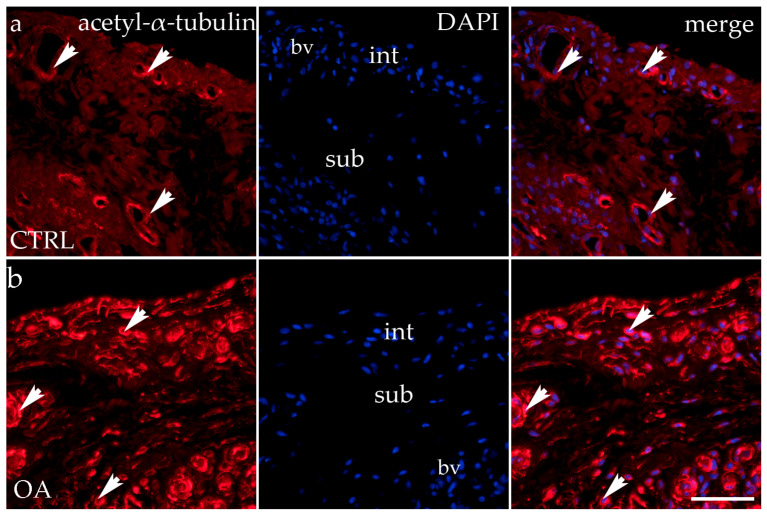
Immunohistochemical staining with acetyl-α-tubulin in the synovial membrane of participants with hip osteoarthritis (OA). (**a**) Hip synovium of participants without OA (controls); (**b**) hip synovium of participants with OA; int—intima; sub—subintima; bv—blood vessel. Acetil-α-tubulin-positive cells (red signal) can be seen in the intima (arrows) and subintima (arrowss) of all analyzed groups (**a**,**b**). Acetil-α-tubulin strong positivity can be seen in cells of the subintimal blood vessels of the control group. All cell nuclei represented in blue are stained with 4′,6-diamidino-2-phenylindole (DAPI). The far-right column (merge) represents acetyl-α-tubulin merged with DAPI nuclear staining. Photos were taken at a magnification of ×40; the scale bar referring to all images is 50 μm. The study included 34 participants, of whom 10 were assigned to the control group and 24 to the osteoarthritis (OA) group.

Acetyl-α-tubulin demonstrated positivity in the intima and subintima of the synovial membrane of participants with hip OA and healthy controls (Figure 5). Strong positivity was noticed in the subintimal blood vessels of all groups analyzed. Statistical analysis showed a significantly higher immunoexpression of acetyl-α-tubulin in the intima compared to the subintima of the control group (*p* < 0.0001), and the same pattern was found in the OA group (*p* < 0.05). The intima and subintima of the OA group had greater positivity (*p* < 0.0001) compared to the intima and subintima of the control group. Acetyl-α-tubulin expression was the lowest in the control group’s intima and subintima. Immunoexpression increased in the intima and the subintima of the OA group (Figure 3).

### 3.6. Differential Gene Expression

**Figure 6:**
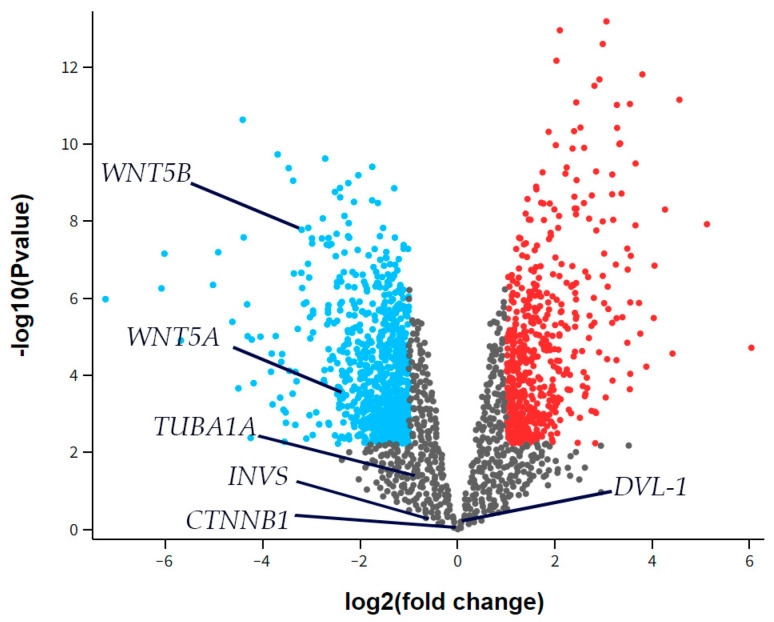
A volcano plot for synovial tissue from healthy joints (CTRL) and osteoarthritis (OA) patients showing differentially expressed genes in the dataset GSE55235. The *x*-axis represents the base 2 logarithm of the fold change, while the *y*-axis represents the negative base 10 logarithm of the false discovery rate. Each dot on the plots represents a gene. All genes with a –log (false discovery rate) > 2, which corresponds to *p* < 0.01, are considered significantly differentially expressed and their dots are colored, while the other genes’ dots are black and are not considered differentially expressed. The red dots on the right represent significantly up-regulated genes, while blue dots are considered significantly down-regulated. The positions of Inversin (*INVS*), Dishevelled-1 (*DVL-1*), α-tubulin (*TUBA1A*), WNT Family Member 5a (*WNT5A*), and WNT Family Member 5b (*WNT5B*) are marked. There was no significant difference in *INVS*, *DVL-1*, or *TUBA1A* expression between the synovial tissue of healthy joints (CTRL) and those with osteoarthritis (OA). However, *WNT5A* and *WNT5B* were significantly down-regulated in OA compared to the control.

The RNA sequencing (RNAseq) data from the GSE55235 series (Rheumatoid arthritis and osteoarthritis: synovial tissues, Berlin dataset) was analyzed to identify any differential expression of *Inversin* (*INVS*), *Dishevelled-1* (*DVL-1*), *α-tubulin* (*TUBA1A*), *WNT Family Member 5a* (*WNT5A*), or *WNT Family Member 5b* (*WNT5B*) between the groups studied, considering a two-fold change as significant. There was no significant difference in the expression of *INVS*, *DVL-1*, or *TUBA1A* between the synovial tissue of healthy joints (CTRL) and those with osteoarthritis (OA) (Figure 6). However, *WNT5A* and *WNT5B* were significantly down-regulated in OA compared to the control.

## 4. Discussion

Our research aimed to investigate the expression and role of a group of functionally related proteins important in bone development and inflammatory conditions, including the proteins of WNT Family Member 5A/B (WNT5A/B), β-catenin, acetyl-α-tubulin, Dishevelled-1 (DVL-1), and Inversin (INV), as potential synovial inflammation moderators in the hip synovium of participants with OA and to compare the expression of these proteins with participants in a controlled group that did not suffer from OA.

The mean value for Krenn was 2 in the range from 0 to 6, while in our study, the mean value was 2.69 in the range from 0 to 6. The difference can be explained by a larger sample in Krenn’s research (n = 212 in Krenn’s research and n = 24 in our study) [58,59].

Early synovial membrane inflammation is believed to play a major role in the patho-physiology of OA, contributing to further inflammation, tissue destruction, and very likely increased expression of the WNT signaling pathway in synovial tissue. For example, cytokine interleukin-1β (IL-1β) induces the expression of WNT5A in chondrocytes and tumor necrosis factor α (TNF-α) induces the expression of WNT5A in the synovium [60,61,62,63,64,65,66,67].

The expression of proteins observed in our study is more prominent in the intima than in the subintima of the synovial membrane, except for WNT5A/B, which is more expressed in the subintima. WNT5A signaling is associated with some of the main features of OA, namely chondrocyte hypertrophy, which is involved in the pathogenesis of OA [68]. Our immunohistochemistry (IHC) results, indicating no significant difference in WNT5A expression between the osteoarthritis (OA) and control groups, align with findings from other studies. Bosch et al. reported no statistically significant difference in WNT5A expression in knee synovial tissue that would correlate with an increased frequency of OA-like cartilage lesions compared to the control group [69]. WNT5A and WNT5B were significantly down-regulated in OA compared to the control in the differential analysis of the GEO dataset. However, it is important to note that WNT5A expression may vary in patients with rheumatoid arthritis (RA). The observed discrepancy between GEO dataset findings, which indicate the down-regulation of WNT5A/B mRNA in osteoarthritis (OA), and IHC results showing unchanged protein levels is likely due to tissue heterogeneity and compartmentalization. The stability of WNT 5A/5B protein expression, despite reduced mRNA levels, is due to complex regulatory mechanisms involving transcription factors, microRNAs (miRNAs), and post-transcriptional modifications. Transcription factors like NF-kB modulate gene expression, while miRNAs such as miR-155 stabilize mRNA and enhance translation. Post-translational modifications, especially phosphorylation, enhance protein production even with lower mRNA levels, and its activity in cellular signaling protects the protein from degradation, extending its half-life. These mechanisms regulate protein interactions, localization, and activation, fine-tuning WNT 5A/5B’s roles in various physiological and pathological processes and offering potential therapeutic strategies in conditions with dysregulated WNT signaling [32,70,71,72]. Spatial variations in synovial WNT5A/B expression, particularly its higher abundance in the subintima, may dilute compartment-specific changes in bulk analyses. Furthermore, mixed cell populations within the synovium could obscure mRNA down-regulation in specific cellular subsets when averaged across all cells in IHC. Sen et al. demonstrated that WNT5A is significantly overexpressed in the synovial tissue of RA patients compared to OA patients and healthy controls [24]. Similarly, Imai et al. found a higher WNT5A expression in the synovium of RA patients than in those with OA [73]. Given these findings, future research should further investigate the role of WNT5A in the progression of RA, as its differential expression may be implicated in its pathogenesis. Understanding the molecular mechanisms underlying WNT5A signaling in RA could contribute to identifying potential therapeutic targets for disease modulation and treatment.

Our findings confirm that β-catenin is more expressed in the intima than in the subintima in both groups, with significantly higher expression in the subintima of the OA group compared to the control group. These results align with previous studies demonstrating increased β-catenin expression in the synovial tissue of OA patients [74,75]. Future research should focus on the role of β-catenin in OA progression, particularly its dynamics across different disease stages. Huang et al. reported higher β-catenin expression in the pre-OA group than in the OA group, suggesting that its expression may play a crucial role in the early stages of the disease [26]. Furthermore, studies such as that of XI et al. highlight the connection between the WNT/β-catenin signaling pathway and inflammatory cytokines, particularly IL-1β, which may contribute to a vicious cycle of inflammation and cartilage degradation [76]. Further investigations should explore the precise regulatory mechanisms of β-catenin in different layers of synovial tissue and its association with inflammatory processes and cartilage destruction. Understanding these mechanisms could aid in developing targeted therapies to modulate the WNT/β-catenin signaling pathway to slow OA progression. According to some studies, the activation of WNT/β-catenin signaling positively affects the repair of cartilage deficits [38,77]. The β-catenin signaling pathway plays a crucial role in promoting the proliferation and activation of synovial fibroblasts, contributing to the development of osteophytes and fibrosis. In our study, the increased expression of β-catenin in the subintima of the OA group, compared to the control group, can be explained by the localization of synovial fibroblasts within the subintima [38,77]. Based on our findings, we assume that the overexpression of β-catenin observed in this study reflects its critical role in cartilage repair and the proliferation of synovial fibroblasts, both of which contribute to the pathological changes characteristic of osteoarthritis (OA).

β-catenin is expressed more strongly in the intima of all groups than in the subintima. It is significantly more expressed in the subintima of the LSS and HSS than in the subintima of the control group and the intima of the LSS than in the intima of the control group. Huang et al. showed a higher expression of β-catenin protein and mRNA in the knee synovium in the pre-OA group than in the OA group, which corresponds to the results of our research, with a higher protein expression in the LSS group than in the HSS group [26]. Wang et al. showed higher β-catenin protein and gene expression in the knee synovium of patients in the OA group than in the control group [74]. Yuan et al. also demonstrated higher protein expression in the OA group than in the control group [75]. These studies confirm the results obtained from our research and the results from the differential analysis. The elevated expression in the LSS group suggests that β-catenin’s protective and reparative functions may be more pronounced in the early stages of OA, while its involvement in promoting inflammation and tissue destruction becomes increasingly significant as the disease advances.

So far, no direct link between synovial inflammation in OA and acetyl-α-tubulin expression has been established. However, it is known that acetyl-α-tubulin expression increases in synovial mesenchymal stem cells treated with docetaxel [78]. In our study, acetyl-α-tubulin showed the highest expression in the OA group, with a statistically significant increase from the control to the OA group in both the intima and subintima. The only known connection between OA and acetyl-α-tubulin in the literature involves histone deacetylase 6 (HDAC6), which was overexpressed on chondrocytes in a mouse OA model and linked to mitochondrial dysfunction and increased ROS production. Reduced HDAC6 activity may lead to higher tubulin acetylation, microtubule stabilization, and enhanced cellular mechanotransduction. In RA, HDAC6 inhibition induces tubulin hyperacetylation in fibroblast-like synoviocytes (FLS), reducing inflammatory cytokine and tissue-degrading enzyme production [79]. Zheng et al. reported that HDAC6 activity disrupts mitochondrial arrangement, leading to ECM degradation and ROS production, an effect counteracted by tubastatin A in a mouse OA model [50]. Thus, the increased expression of acetyl-α-tubulin in the OA group in our study may represent a compensatory response to synovial inflammation or cellular stress, aiming to stabilize the cytoskeleton and maintain cellular function in degenerative conditions.

DVL-1 plays a key role in cellular processes and signaling pathways that regulate cartilage integrity and homeostasis [80,81]. Mutations in DVL-1 can cause Robinow syndrome, which is characterized by bone abnormalities, highlighting its importance in bone function [80]. Elevated DVL-1 levels can hyperactivate the WNT/β-catenin signaling pathway, leading to cartilage degradation and abnormal bone remodeling, both typical features of OA [81]. In our study, DVL-1 expression was significantly higher in the OA group compared to the healthy control group. Although there is no direct evidence linking DVL-1 to synovial inflammation in OA, its role in RA, where it supports the survival of fibroblast-like synoviocytes (FLS), suggests a potential indirect influence on the inflammatory processes within OA joints [82].

INV is primarily involved in regulating primary cilia function and cell polarity. Although various studies have linked the WNT signaling pathway and INV with cilia dysfunction in OA, the precise mechanism of this association remains unclear. The expression of INV in our study and in the differential analysis of the GEO dataset did not differ significantly between the osteoarthritis (OA) and control groups, suggesting that INV may not play a substantial role in synovitis associated with OA. However, Brya et al. suggested that INV might reduce synovial membrane inflammation by acting as a molecular switch between different WNT signaling cascades, targeting DVL-1 for degradation via the ubiquitin–proteasome pathway, thereby inhibiting the canonical WNT pathway [42,43,44,45,83]. INV’s role in osteoarthritis (OA) may be more closely associated with maintaining cellular architecture or regulating signaling pathways rather than directly contributing to synovial inflammation. This may account for our study’s observed lack of differential expression between the OA and control groups. By expanding the study, we did not find any significant differences in INV expression considering OA severity.

The control group in this study included participants without osteoarthritis (OA) who underwent hip surgery due to trauma rather than elective procedures for tissue collection, which would be challenging due to ethical constraints, especially in younger individuals, which could be solved by using animal models. Additionally, trauma patients may already show mild OA changes, affecting the outcomes. Limitations include a small sample size for synovial membrane sampling and the lack of advanced techniques like Western blotting. Histopathological scores for synovitis also vary by biopsy location and between primary and secondary OA. Future research should refine control group selection, increase sample size, incorporate advanced techniques, and explore histopathological variability and its clinical significance.

## 5. Conclusions

Our findings suggest that WNT5A/B and β-catenin play a key role in synovial inflammation and the progression of OA. WNT5A/B and β-catenin levels may serve as biomarkers to assess disease severity, predict progression, or evaluate treatment responses, aiding personalized treatment approaches. Modulating WNT5A/B could offer new treatments to reduce inflammation, slow OA, and preserve joint function. WNT pathway inhibitors such as XAV-939 and SM04690 also have impact on reducing β-catenin activity and have shown promise in both preclinical and clinical trials [84,85].

Our research has shown that altered acetylation patterns of acetyl-α-tubulin affect microtubule stability in osteoarthritis (OA) and contribute to degenerative processes in cartilage and synovial tissue. This makes it a potential biomarker for assessing the progression of OA. Additionally, it could also serve as a biomarker for evaluating treatment response, as the experimental drug tubastatin A has been shown to be effective in treating murine OA, provided that the same effect is confirmed in humans. However, further in-depth studies are essential to clarify therapeutic potential [48,49,50].

## Figures and Tables

**Table 1:** Antibodies used for immunofluorescence.

Antibodies		Host	Dilution	Source
Primary	Anti-Acetyl-A-Tub/12152S	Mouse	1:500	Cell Signaling Technology (CST), (Danvers, MA, USA)
	Anti-Inversin/ab65187	Rabbit	1:100	Abcam (Cambridge, UK)
	Anti-DVL1/sc8025	Mouse	1:50	Santa Cruz Biotechnology (Dallas, TX, USA)
	Anti-Wnt5a/b/2530S	Rabbit	1:100	Cell Signaling Technology (CST), (Danvers MA, USA)
	Anti-β-catenin/2677S	Mouse	1:200	Cell Signaling Technology (CST), (Danvers, MA, USA)
Secondary	Anti-Rabbit IgG, Alexa Fluor^®^ 488, 711-545-152	Donkey	1:300	Jackson Immuno Research Laboratories, Inc., (Baltimore, PA, USA)
	Anti-Mouse IgG, Rhodamine Red™-X, 715-295-151	Donkey	1:300	Jackson Immuno Research Laboratories, Inc., (Baltimore, PA, USA)

**Table 2:** Clinical, radiological, and pathohistological characteristics of the examined groups (*n* = 34).

	Age (Median ± IQR, Years)	Sex (Male/Female)	BMI (Median ± IQR, kg/m^2^)	K-L Grade (Median ± IQR)	Krenn Score (Median ± IQR)	HHS (Median ± IQR)	VAS (Median ± IQR)	WOMAC (Median ± IQR)
Controls	74 (73.55–76.05)	(6/4)	25.87 (23.97–26.6)	0.5 (0–1)	0 (0–0)	-	-	-
OA Krenn Synovitis Score 0–2	73 (63.7–75.9)	(7/5)	24.7 (23.25–25.82)	2 (2–2)	6.4 (5.6–9)	48.7 (43.58–56.8)	6 (4.6–6.8)	46.2 (40.2–56.4)
OA Krenn Synovitis Score ≥ 3	73 (66–78)	(6/6)	26.7 (25.5–29.43)	4 (3–4)	9 (7–9)	41 (33.48–49.6)	6 (5–7)	47.3 (36.1–55.3)
* *p* value	0.854	0.732	0.054	<0.0001	<0.0001	0.272	0.784	0.918

IQR (interquartile range), OA (osteoarthritis), BMI (body mass index), K-L grade (Kellgren–Lawrence grading scale), HHS (Harris Hip Score), VAS (visual analogue scale), WOMAC (The Western Ontario and McMaster Universities Osteoarthritis Index); * *p* < 0.05, Kruskal–Wallis test.

## Data Availability

Data will be available upon request.

## Supplementary Materials

The following supporting information can be downloaded at: https://www.mdpi.com/article/10.3390/biomedicines13040995/s1. Figure S1. Statistical analyses of protein immunoexpression of Inversin (INV) (a), β-catenin (b) in the synovial membrane of participants with hip osteoarthritis (OA). INT—intima, SUB—subintima, CTRL—controls, LSS—low synovitis score of OA (Krenn score 0–2), HSS—higher synovitis score of OA (Krenn score ≥ 3). We analyzed the data using two-way ANOVA with Tukey’s post hoc test. The bars of the graphs represent the mean area percentage of the immunofluorescence signal of the analysed proteins, while the error bars represent the standard deviation. Asterisks mark significant differences: * *p* < 0.05, *** *p* < 0.001.
