# Supplementary material for: WNT Signaling Factors as Potential Synovial Inflammation Moderators in Patients with Hip Osteoarthritis
Source: Biomedicines. 2025 Apr 19;13(4):995. doi: 10.3390/biomedicines13040995 (PMC12025112; doi:10.3390/biomedicines13040995)
Supplement: Supplementary file 1 [file biomedicines-13-00995-s001.zip › biomedicines-3553154-supplementary.pdf]

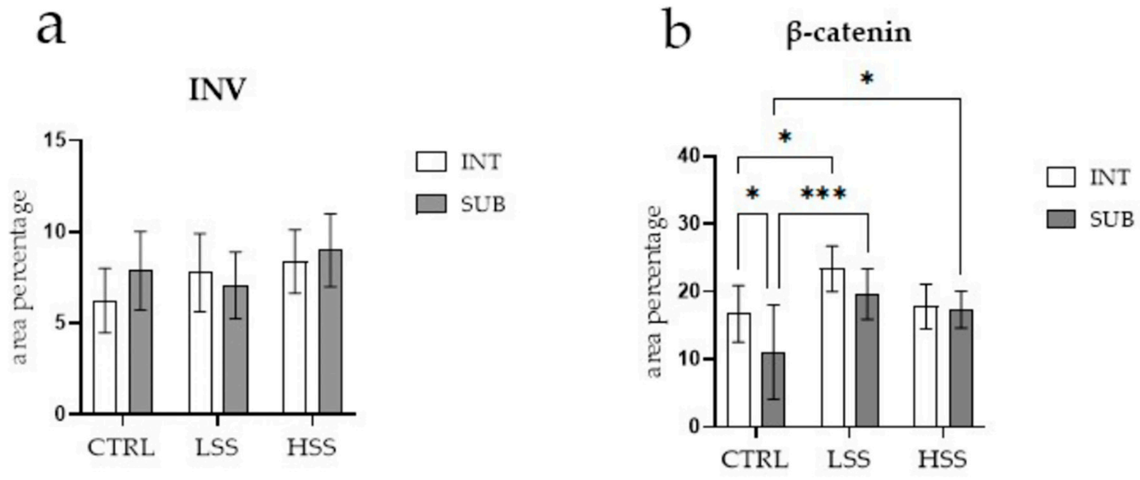

Figure S1. Statistical analyses of protein immunoexpression of (a) Inversin (INV) and (b) β-catenin in the synovial membrane of participants with hip osteoarthritis (OA). INT—intima, SUB—subintima, CTRL—controls, LSS—low synovitis score of OA (Krenn score 0–2), HSS—higher synovitis score of OA (Krenn score ≥ 3). We analyzed the data using two-way ANOVA with Tukey's post hoc test. The bars of the graphs represent the mean area percentage of the immunofluorescence signal of the analysed proteins, while the error bars represent the standard deviation. Asterisks mark significant differences: * $p < 0.05$, *** $p < 0.001$.
